# Incidence of anti-platelet factor4/polyanionic antibodies, thrombocytopenia, and thrombosis after COVID-19 vaccination with ChAdOx1 nCoV-19 in Thais

**DOI:** 10.1186/s12959-023-00533-z

**Published:** 2023-09-06

**Authors:** Kochawan Boonyawat, Tichayapa Phojanasenee, Phichchapha Noikongdee, Pornnapa Police, Pichika Chantrathammachart, Pimjai Niparuck, Teeraya Puavilai, Angsana Phuphuakrat, Pantep Angchaisuksiri

**Affiliations:** Department of Medicine, Faculty of Medicine Ramathibodi Hospital, Mahidol University, 270 Rama 6th Road, Rachathewi, Bangkok, 10400 Thailand

**Keywords:** COVID-19 vaccines, Incidence, Anti-PF4/polyanionic antibodies, Thai

## Abstract

**Background:**

The prevalence of anti-platelet factor 4 (PF4)/polyanionic antibodies occurring after vaccination with ChAdOx1 nCoV-19 is low. Most of these antibodies are not associated with vaccine-induced thrombotic thrombocytopenia. It remains unknown whether these antibodies are preexisting or occur as a result of vaccination. In this study, we demonstrated the incidence of anti-PF4/polyanionic antibodies, thrombocytopenia, and thrombosis after vaccination with ChAdOx1 nCoV-19 in a large cohort of Thais.

**Methods:**

We conducted a prospective study in a cohort of health care workers and members of the general population who received COVID-19 vaccination with ChAdOx1 nCoV-19. Blood collection for complete blood count, D-dimer, and anti-PF4/polyanionic antibodies was performed before vaccination (day 0), day 10, and day 28 after vaccination. Anti-PF4/polyanionic antibodies were detected using enzyme-link immunosorbent assay (ELISA). Functional assay was performed for all positive ELISA tests.

**Results:**

A total of 720 participants were included in the study. 214 participants received both the first and second doses, 91 participants received only the first, 51 received only the second, and 364 received the third booster dose of ChAdOx1 nCoV-19. Median age was 42 years (IQR, 34–53). 67% of participants were female. Three participants developed seroconversion, yielding an incidence of vaccination-induced anti-PF4/polyanionic antibodies of 0.42% (95% confidence interval 0.08, 1.23). Fourteen (1.9%) participants had preexisting anti-PF4/polyanionic antibodies before the vaccination but their optical density of anti-PF4/polyanionic antibodies did not significantly increase over time. None of the anti-PF4/polyanionic positive sera induced platelet aggregation. Abnormal D-dimer levels following vaccination were not different among the positive and negative anti-PF4/polyanionic groups (11.8% vs. 13.2%, p = 0.86). Thrombocytopenia occurred in one person with negative anti-PF4/polyanionic antibodies. No clinical thrombosis or bleeding occurred.

**Conclusion:**

We found a low incidence of seroconversion of anti-PF4/polyanionic antibodies after vaccination with ChAdOx1 nCoV-19 in Thais. Most of the anti-PF4/polyanionic antibodies were preexisting and did not significantly increase after vaccination with ChAdOx1 nCoV-19. Following vaccination, some participants with anti-PF4/polyanionic antibodies had elevated D-dimer levels, while only one developed thrombocytopenia and no thrombotic events were observed.

## Introduction

Vaccine-induced thrombocytopenia (VITT) has been an emerging condition since 2020 after ChAdOx1 nCoV-19 vaccination. It also has been reported occurring after vaccination with mRNA vaccine [1]. The pathogenesis of VITT is still unclear but relates to the generation of anti-platelet factor 4/polyanionic antibodies resembling those seen in heparin induced thrombocytopenia [2]. We previously demonstrated a low prevalence of anti-PF4/polyanionic antibodies occurring after vaccination with CoronaVac and ChAdOx1 nCoV-19 among health care workers [3]. Whether these antibodies are preexisting or a result of vaccination with ChAdOx1 nCoV-19 has not been elucidated. A study from Germany reported that 2 of 11 available samples had seroconversion of anti-PF4/polyanionic antibodies after ChAdOx1 nCoV-19 vaccination [4]. In the Asian population, the incidence of VITT is lower than in Caucasians [5]. In this study we conducted a prospective cohort study on the incidence of anti-PF4/polyanionic antibodies, thrombocytopenia, and thrombosis after vaccination with the first, second or the third booster doses of ChAdOx1 nCoV-19.

## Materials and methods

### Study population and settings

A prospective cohort study was conducted during May 12st, 2021 to November 30th 2021 at Ramathibodi Hospital, a tertiary care academic hospital in Bangkok, Thailand. Adult health care workers and volunteer participants who received the first and/or second dose of the ChAdOx1 nCoV-19 vaccine were included in the study. We also included health care workers who received the third booster dose with ChAdOx1 nCoV-19 (having previously received 2 doses of CoronaVac) since this group also had first exposure to ChAdOx1 nCoV-19. The time intervals between the first and second doses of ChAdox1 vaccines and between the second dose of CoronaVac and third dose of ChAdox1 vaccine were 3 months. All participants gave written informed consent. The study protocol was approved by the Human Research Ethics Committee of the Faculty of Medicine at Ramathibodi Hospital, Mahidol University.

### Questionnaires

After informed consent, participants were asked to complete questionnaires at baseline prior to each vaccination, on day 10, and on day 28 after vaccination. Questions included baseline characteristics, age, sex, comorbidities, previous thrombosis, use of hormonal therapy, family history of thrombosis, and current oral antithrombotics. At day 10 and day 28 after the vaccination, participants were asked about adverse effects and symptoms suggestive of thrombosis or bleeding, and a history of recent COVID-19 infection.

### Blood collection and laboratory analysis

Blood collection for complete blood count (CBC), D-Dimer, and anti-PF4/polyanionic antibodies were drawn from participants prior to the vaccination to day − 3, day 10 ± 3, and day 28 ± 3. Complete blood count was determined using Sysmex. D-dimer levels were determined by Innovance D-Dimer microparticle-enhanced immunoassay (Dade Behring). Results for D-dimer levels were reported in ng/mL FEU. The normal level was less than 500 ng/mL FEU. Anti-PF4/polyanionic antibodies were screened by IgG-specific ELISA (Hyphen Biomed Zymutest HIA IgG, Quadratech Diagnostics, UK) according to the manufacturer’s instructions. Results were interpreted as positive if the optical density (OD) was above 0.3. Positive samples in ELISA were tested by platelet aggregation on the CHRONO-LOG® platelet aggregometer (Chrono-log Corporation, PA, USA). Normal blood group O donor platelets were incubated with PF4/polyanion-positive sera in the presence of low-dose heparin (unfractionated heparin 1.0 IU/mL), high-dose heparin (unfractionated heparin 100 IU/mL), or saline buffer. A previously confirmed HIT serum was used as a positive control and normal pooled plasma as a negative control.

### Statistical analysis

Baseline characteristics in continuous variables were analyzed and presented with mean and standard deviation or median and interquartile range as appropriate. Categorical variables were presented in percentages. Multilevel mixed-effects linear regression was performed to compare the results of anti-PF4 antibodies and D-Dimer overtime. All statistical analyses were performed on GraphPad Prism 9.1.1 (GraphPad Software, CA, USA) and Stata statistical software version 15.1 (StataCorp, TX, 2018).

## Results

A total of 720 participants receiving the first, second, or third booster dose of ChAdOx1 nCoV-19 were included in the study. There were 214 participants who received both first and second doses of the vaccine. 91 participants received only the first dose of the vaccine. 51 participants received only the second dose of the vaccine. 364 participants received only the third booster dose of the vaccine. Median age of all participants was 42 years (IQR, 34–53). 67% of participants were female. Overall, 26 participants were on antithrombotics. Three participants received anticoagulants (2 warfarin and 1 dabigatran) for the indication of treatment of deep vein thrombosis and prevention of systemic thromboembolism in atrial fibrillation. 23 participants received antiplatelets (aspirin and/or clopidogrel) for primary prevention and for treatment of coronary artery disease. Baseline characteristics and laboratory results are presented in Tables 1 and 2, respectively.

**Table 1:** Baseline characteristics of study subjects

Characteristics	Type of vaccine			
	First ChAdOx1 only or first and second ChAdOx1 (n = 305) *	Second ChAdOx1 only (n = 51) **	First and second CoronaVac/ third ChAdOx1 (n = 364) ***	
Age, years (median, IQR)	51 (40–61)	39 (31–45)	39 (32.5–45)	
Female, n (%)	167 (54.8)	20 (39.2)	50 (13.7)	
Hormonal use, n (%)	18 (5.9)	3 (5.8)	29 (8)	
Heparin exposure within 3 months, n (%)	2 (2.2)	0	1 (0.3)	
Antithrombotics, n (%)	17 (5.6)	2 (3.9)	7 (1.9)	
Family history of thrombosis, n (%)	11 (3.6)	0	7 (1.9)	

* 91 participants received only first dose of ChAdOx1 and 214 participants received both first and second dose of ChAdOx1.

** All participants previously exposed to the first dose of ChAdOx1 but did not participate in the study at that time

*** Since these participants had first exposure to ChAdOx1, they were included in the study

**Table 2:** Laboratory results at various time points

Characteristics	Type of vaccine								
	First dose ChAdOx1 (n = 305)*			Second dose ChAdOx1 (n = 265)**			CoronaVac/CoronaVac/ChAdOx1 (n = 364)***		
	**Day 0** (n = 304)	**Day 10** (n = 278)	**Day 28** (n = 275)	**Day 0** (n = 264)	**Day 10** (n = 240)	**Day 28** (n = 249)	**Day 0** (n = 364)	**Day 10** (n = 333)	**Day 28** (n = 336)
PF4/polyanion EIA +, n (%)	5 (1.6)	4 (1.4)	4 (1.5)	7 (2.7)	7 (2.9)	7 (2.8)	6 (1.7)	6 (1.8)	5 (1.5)
D-dimer, ng/L FEU (median, IQR)	270 (165–410)	308 (200–510)	270 (160–440)	235 (140–380)	230 (140–380)	210 (140–350)	245 (170–370)	260 (180–380)	240 (160–370)
Platelet count, x 10^9^/L (mean, SD)	272 (64.3)	273 (65.9)	272 (66.4)	264 (63.0)	282 (63.7)	275 (64.5)	301 (64.5)	324 (70.0)	298 (66.7)

* 91 participants received only first dose of ChAdOx1 and 214 participants received both first and second dose of ChAdOx1.

** 214 participants received both first and second dose of ChAdOx1 and 51 participants received only second dose of ChAdOx1.

*** Since these participants had first exposure to ChAdOx1, they were included in the study

**Fig. 1:**
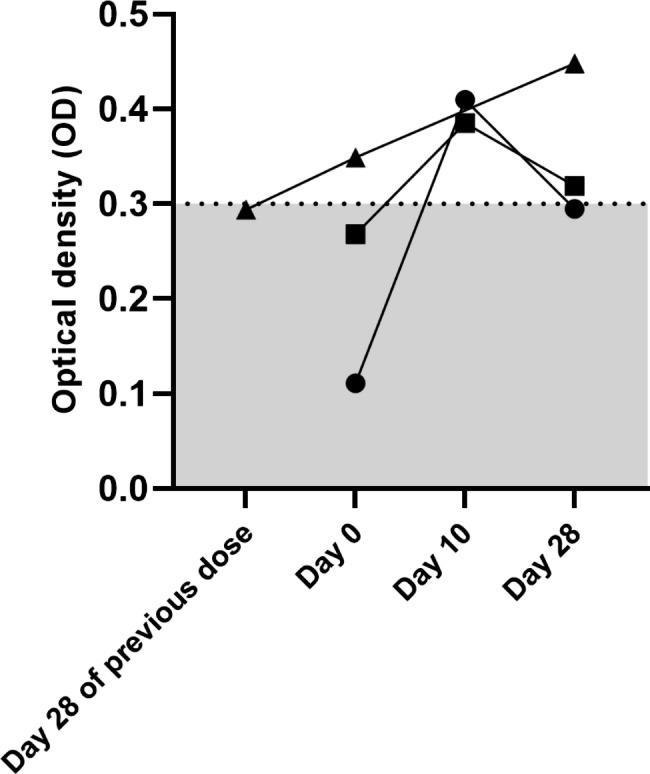
Optical density of 3 samples with negative anti-PF4/polyanionic antibodies at baseline and conversion to positive at various time points. One participant developed seroconversion at day 0 of the second dose (Triangle). Two participants had seroconversion at day 10 after the second (square) and the booster dose (circle), respectively. Grey shading indicates negative result

Overall, 3 participants developed seroconversion. Therefore, the incidence of anti-PF4/polyanionic antibodies was 0.42% (95% confidence interval [CI] 0.08, 1.23). One participant developed seroconversion after the second dose of ChAdOx1 nCoV-1, while the previous results after the first dose were negative. Two participants had seroconversion at day 10 after the second dose of ChAdOx1 nCoV-1 and the third booster dose, respectively. All seroconverted antibody levels were modest (OD 0.3–0.5). In the 2 participants with seroconversion at day 10, the antibodies were transient, becoming negative by day 28 (Fig. 1).

**Fig. 2:**
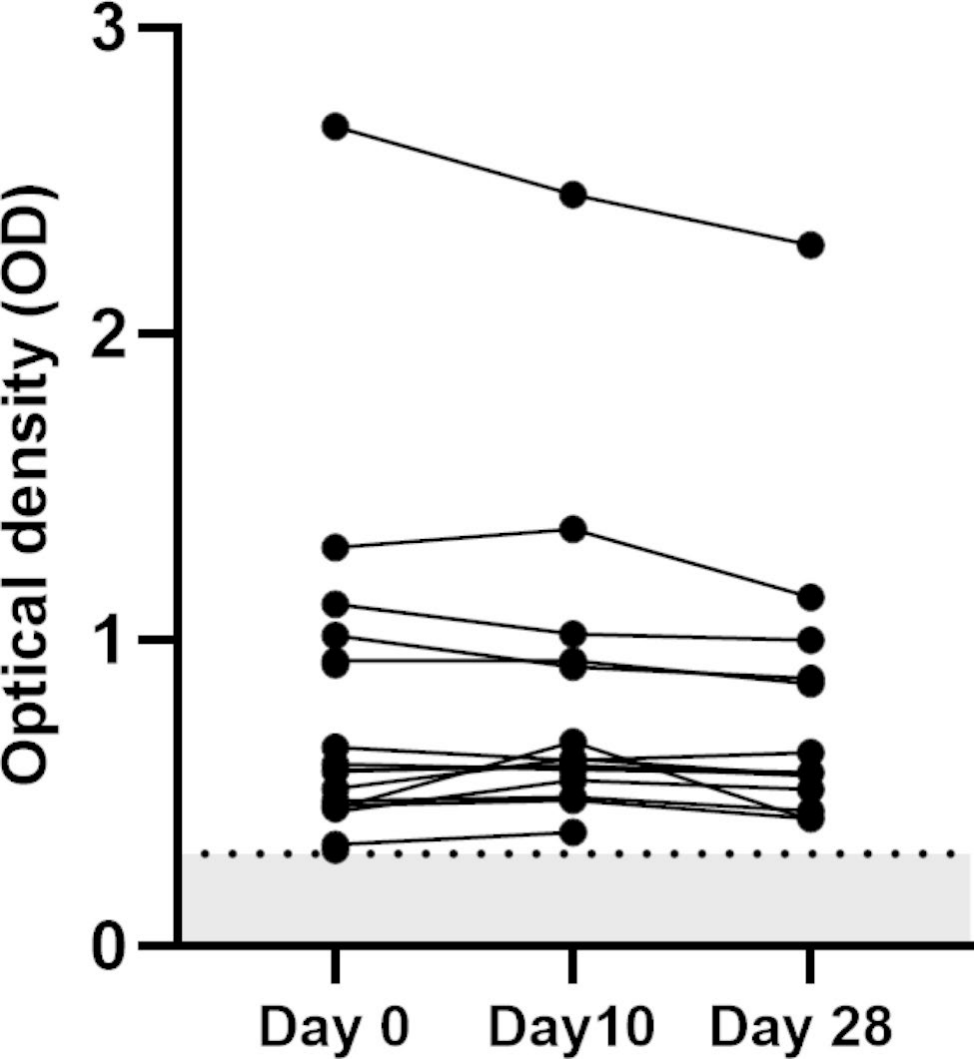
Optical density of 14 samples with positive anti-PF4/polyanionic antibodies at baseline that were persistently positive on follow up. Grey shading indicates negative result

**Fig. 3:**
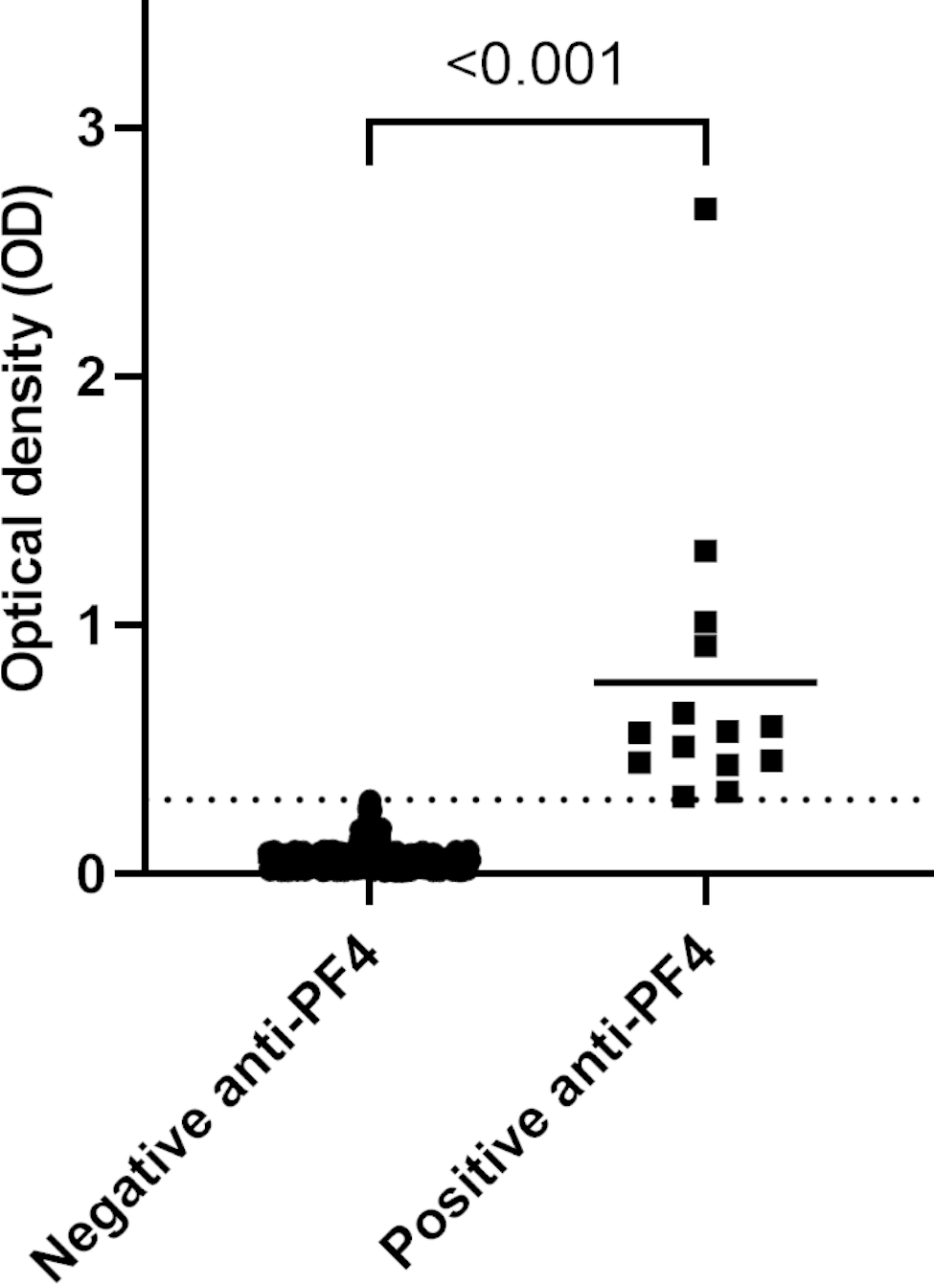
Optical density of 14 samples with positive and 704 samples with negative anti-PF4/polyanionic antibodies at baseline (before vaccination). Horizontal lines in the positive and negative sera column represent mean OD. Dot line represents the positive cutoff OD value of 0.3

Fourteen participants had preexisting antibodies; thus, the prevalence of anti-PF4/polyanionic antibodies was 1.94% (95% CI 1.07, 3.24). Mean OD of the preexisting positive antibodies was 0.77 (standard deviation [SD] 0.61). The minimum and maximum ODs were 0.31 and 2.68. The optical density of anti-PF4/polyanionic antibodies did not significantly increase over time (Fig. 2). Mean OD of the negative samples was 0.06 (SD 0.04). The minimum and maximum ODs of negative samples were 0.01 and 0.30. There was a significant difference in mean OD of the positive and the negative samples (p-value < 0.001) (Fig. 3). Of all the positive samples, none of the anti-PF4/polyanionic positive sera induced platelet aggregation. None of the anti-PF4/polyanionic positive participants received antithrombotics.

### D-dimer

**Fig. 4:**
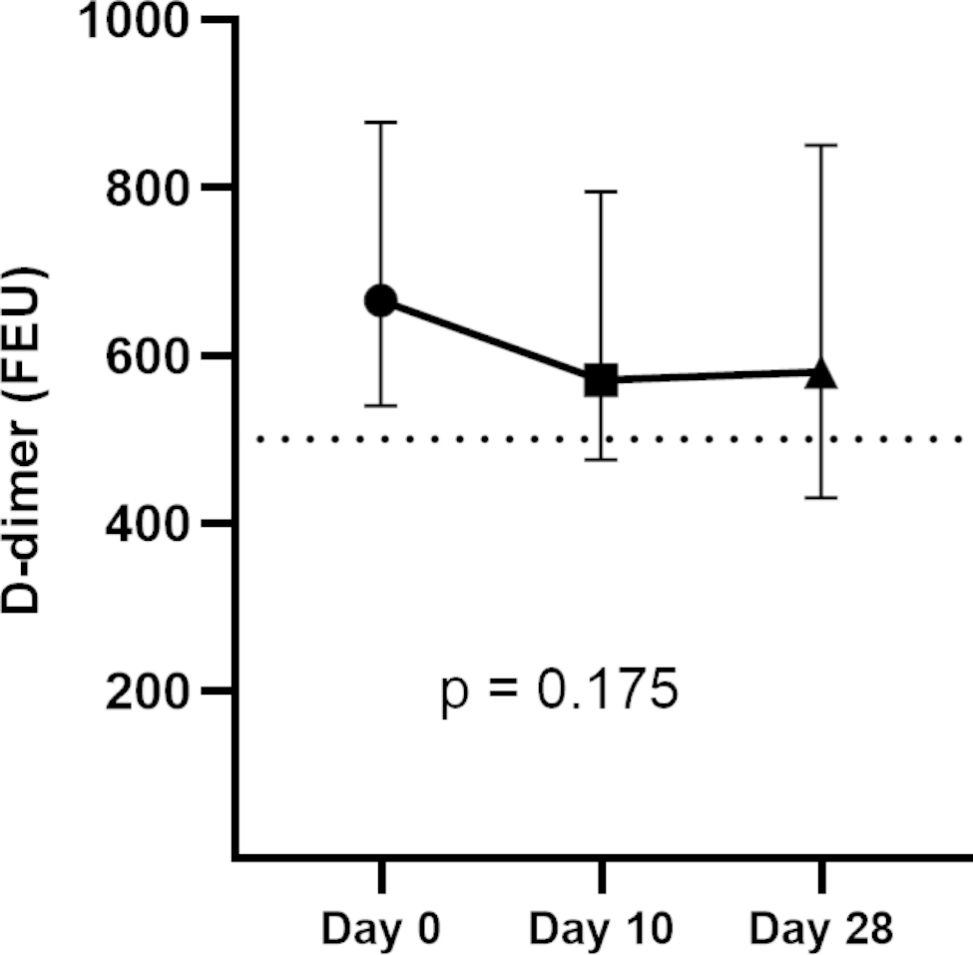
Median D-dimer levels (interquartile range) of those with abnormal D-dimer levels at baseline at subsequent time points (113 samples with abnormal D-dimer levels at day 0, 142 samples at day 10, and 102 samples at day 28). D-dimer levels are presented in fibrinogen equivalent unit (FEU). Dotted line represents the positive cutoff value of 500 FEU

**Fig. 5:**
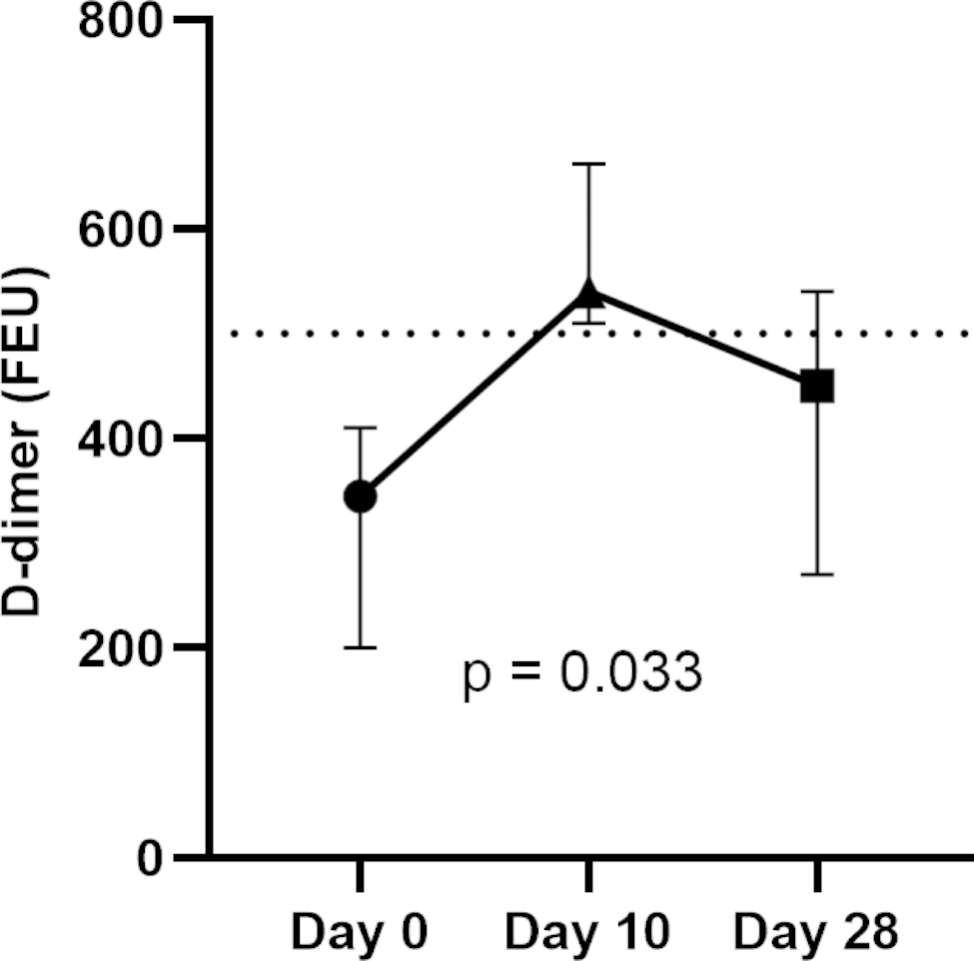
Median D-dimer levels (interquartile range) of those with normal D-dimer level at baseline who became abnormal after vaccination (95 samples at day 0, 87 samples at day 10, and 52 samples at day 28). D-dimer levels are presented in fibrinogen equivalent unit (FEU). Dotted line represents the positive cutoff value of 500 FEU

Overall, 95 (13.2%) participants with normal D-dimer levels at baseline demonstrated an abnormal D-dimer level at day 10 or/and day 28. Preexisting abnormal D-dimer level (> 500 FEU) was observed in 113 (15.7%) participants. In these subjects, D-dimer levels did not significantly increase over time (Fig. 4). However, in those with abnormal D-dimer levels after vaccination, the levels of D-dimers were significantly increased compared with baseline levels (p = 0.03) (Fig. 5).

In those with positive anti-PF4/polyanionic antibodies (including those who seroconverted), there were 2 (11.8%) participants who had abnormal D-dimer levels following vaccination. In those with negative anti-PF4/polyanionic antibodies, 92 (13.2%) had abnormal D-dimer levels following vaccination. There was no significant difference in abnormal D-dimer level following vaccination between anti-PF4 antibodies/polyanionic antibody positive and antibody negative groups (p = 0.86).

### Thrombocytopenia

**Fig. 6:**
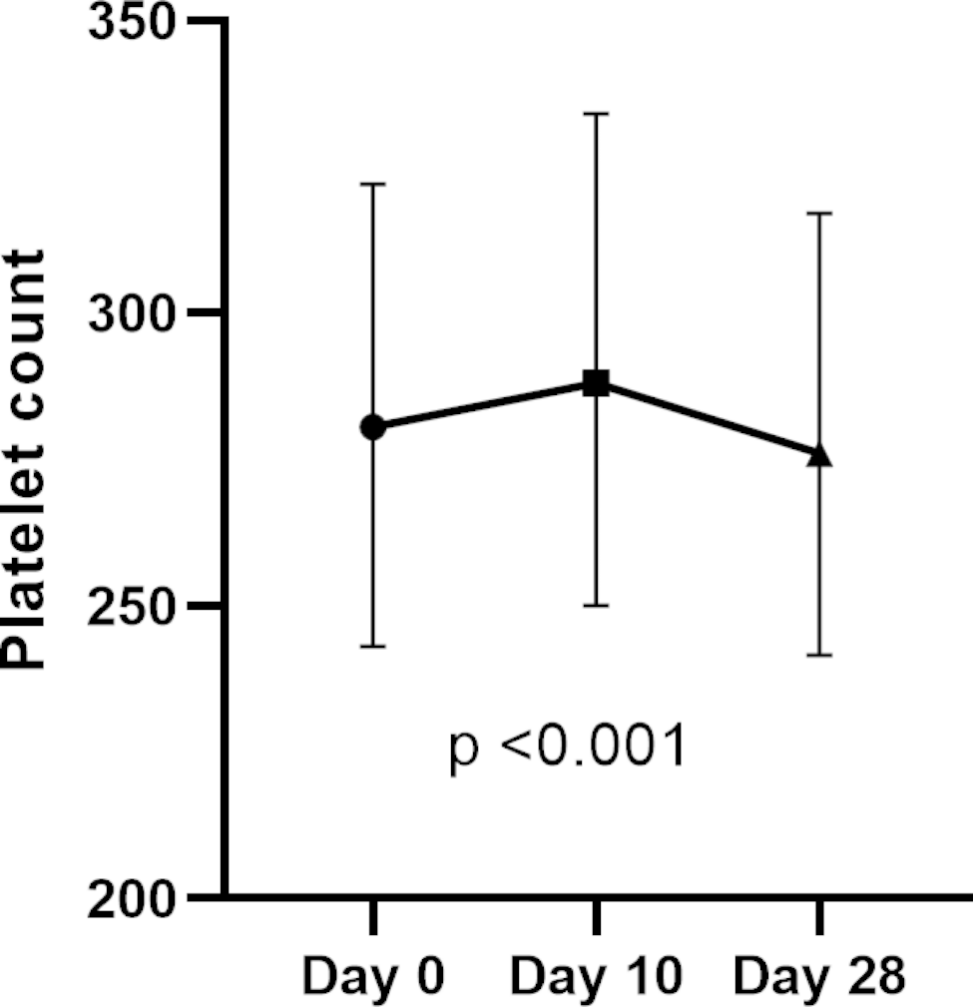
Mean platelet counts (SD) of all participants at various time points. Platelet couts are presented in x10^9^/L

Overall mean platelet count at baseline was 284 × 10^9^/L. On days 10 and 28, mean platelet counts were 294 and 282 × 10^9^/L, respectively (Fig. 6). There was a significant increase of platelet count on day 10, followed by a decrease on day 28 compared with baseline (p < 0.001). One participant developed thrombocytopenia following vaccination on day 28. His baseline platelet count was 181 × 10^9^/L, 153 × 10^9^/L on day 10 and 61 × 10^9^/L on day 28. His platelet count returned to normal prior to the second dose of the vaccine and remained persistently normal. D-dimers were not significantly increased. No clinical thrombosis or bleeding was observed in this or any other participant.

### Symptoms related to vaccination

Overall, 70% of all participants reported having adverse effects after the first exposure to ChAdOx1 nCoV-19. Most common adverse effects were fever and myalgia. 16.7% of participants reported having severe adverse effects after the first exposure. After the second dose of vaccine, adverse effects were reported in 40% of participants. Most of the reported severe adverse effects occurred after the first vaccine exposure. Severe adverse effects were not associated with abnormal D-dimer levels or with the presence of anti-PF4 antibodies.

## Discussion

We report that the incidence of seroconversion after vaccination with ChAdOx1 nCoV-19 is rare, and confirm a low prevalence of anti-PF4/polyanionic antibodies in the general population who received ChAdOx1 nCoV-19 vaccination. We also demonstrated that a number of participants had preexisting antibodies unrelated to the vaccination. Increased D-dimer levels after vaccination could be related to the inflammatory milieu after vaccination. Elevation of D-dimer was likely non-specific as the D-dimer level declined overtime and had no association with the subjectively reported adverse effects of the vaccine. Transient thrombocytopenia occurred in 1 patient. No significant clinical thrombosis or bleeding events were observed.

In this large cohort of Thais who received vaccination with ChAdOx1 nCoV-19, rare seroconversion of anti-PF4/polyanionic antibodies developed. Notably, the antibodies of converted sera were of low titer and transient. Only one participant who had negative anti-PF4/polyanionic antibodies after the first dose of vaccine developed seroconversion after the second dose. In this participant, the OD of anti-PF4 antibodies slightly increased over time. No elevation in D-dimer level, thrombocytopenia or clinical thrombosis developed.

Though the prevalence of anti-PF4/polyanionic antibodies in our study was lower than those reported in Caucasian subjects, the maximum OD observed was 2.675. This number was as high as in patients with VITT. The participant had no underlying disease, no previous heparin exposure and reported few adverse effects after the vaccination. No elevation in D-dimer level, thrombocytopenia, or clinical thrombosis was observed in this participant.

Anti-PF4/polyanionic antibodies have been reported to be natural preexisting antibodies in a minority of the population. In a healthy subject without exposure to heparin, the prevalence of anti-PF4/polyanionic antibodies were reported to be 4.3–6.6% [6, 7]. Most of the reported antibodies were weak antibodies and the OD was rarely above 1. In addition, the prevalence of preexisting anti-PF4/polyanionic antibodies varies by study population, and laboratory assay used to measure. To date, the reports of prevalence and seroconversion of anti-PF4 antibodies following COVID-19 vaccination are summarized in Table 3. In Asians, the prevalence of anti-PF4/polyanionic antibodies following heparin exposure seems to be lower than in western countries. Polymorphism and genetic variants might contribute to the low prevalence of anti-PF4/polyanionic antibodies found in our study [8]. Further studies evaluating the association between genetics and anti-PF4 antibodies/polyanionic antibodies in Asians are warranted.

**Table 3:** Prevalence and incidence of anti-PF4 antibodies following COVID-19 vaccination

Author	Laboratory assay	Prevalence	Seroconversion	Type of vaccine
Ingvild(9)	Lifecodes PF4 IgG ELISA immunoassay (Immucor, Waukesha, WI)	6/492 (1.2%)	Not reported	ChAdOx1
Thiele(4)	In-house IgG- specific PF4/polyanion EIA	11/138 (8%) 8/143 (5.6%)	2/138 (1.4%) 2/143 (1.4%)	ChAdOx1 BNT162b2
Uaprasert(10)	Zymutest HIA IgG (Hyphen Biomed, Neuville-sur-Oise, France)	16/521 (3.1%)	Not reported	ChAdOx1
Noikongdee(3)	Zymutest HIA IgG (Hyphen Biomed Zymutest HIA IgG; Quadratech Diagnostics, Surrey, United Kingdom)	5/221 (2.3%) 4/ 232 (1.7%)	Not reported	ChAdOx1 CoronaVac
Terpos(11)	ELISA (Asserachrom, Stago, Vienna, Austria)	29/43 (67%)	Not reported	ChAdOx1
Hantrakun(12)	Zymutest HIA, IgG ELISA (Hyphen BioMed, Neuville-sur-Oise, France)	10/396 (2.5%)	2/396 (0.5%)	ChAdOx1
In this study	Zymutest HIA IgG (Hyphen Biomed Zymutest HIA IgG; Quadratech Diagnostics, Surrey, United Kingdom)	14 (1.9%)	3/720 (0.42%)	ChAdOx1

We acknowledged the limitations of the study. The number of participants was lower than expected sample size. We were unable to recruit participants after November 2021 due to the change of COVID-19 vaccine in Thailand from ChAdOx1 to mRNA vaccines In addition, although D-dimer levels were significantly elevated following vaccination, we did not collect data on other factors that might affect the D-dimer levels.

In conclusion, we found a low incidence of seroconversion of anti-PF4/polyanionic antibodies after vaccination with ChAdOx1 nCoV-19 in Thais. Most of the anti-PF4/polyanionic antibodies were preexisting and did not significantly increase after vaccination with ChAdOx1 nCoV-19. Some participants with anti-PF4/polyanionic antibodies had elevated D-dimer levels. One episode of thrombocytopenia and no clinical thrombosis were observed.

## Data Availability

Not applicable.
